# Unravelling mitochondrial succinate transport during I/R and devising a novel transport inhibitor show promise for targeting I/R injury, but will efficacy remain under more human-like conditions present during I/R?

**DOI:** 10.1093/cvr/cvag059

**Published:** 2026-03-17

**Authors:** Qian Wang, Xin Hu, Coert J Zuurbier

**Affiliations:** Department of Anesthesiology, Amsterdam Cardiovascular Sciences, Laboratory of Experimental Intensive Care and Anesthesiology, Amsterdam UMC, University of Amsterdam, Meibergdreef 9, Amsterdam, AZ 1105, the Netherlands; Department of Anesthesiology, Amsterdam Cardiovascular Sciences, Laboratory of Experimental Intensive Care and Anesthesiology, Amsterdam UMC, University of Amsterdam, Meibergdreef 9, Amsterdam, AZ 1105, the Netherlands; Department of Anesthesiology, Amsterdam Cardiovascular Sciences, Laboratory of Experimental Intensive Care and Anesthesiology, Amsterdam UMC, University of Amsterdam, Meibergdreef 9, Amsterdam, AZ 1105, the Netherlands

**This editorial refers to ‘Mitochondrial succinate transport is required for cardiac ischaemia/reperfusion injury’, by L. Pala *et al.*, https://dx.doi.org/10.1093/cvr/cvag031.**

The discovery by the Cambridge group that ischaemic accumulation of the TCA-cycle intermediate succinate is a major driver of ischaemia–reperfusion (I/R)–induced ROS production via reverse electron transport (RET) at complex I represents one of the seminal observations elucidating the mechanisms of I/R injury across tissues and organs.^1^ During ischaemia succinate is transported out of the mitochondria, thus allowing continuous production yet it accumulates in the cytosol. Indeed, rapid separation of the mitochondrial and cytosolic compartments demonstrated that succinate predominantly accumulated in the much larger cytosolic compartment during SDH inhibition or anoxia.^2^ An unresolved question in the ischaemic succinate paradigm is the molecular mechanism by which succinate exits the mitochondrial matrix during ischaemia and, conversely, how it re-enters the matrix during early reperfusion to drive RET-mediated ROS production. The inner mitochondrial membrane (IMM) is largely impermeable to most metabolites in order to maintain the proton electrochemical gradient required for oxidative phosphorylation and mitochondrial ATP synthesis. Two mitochondrial carriers have been proposed as candidates for succinate transport:^3^ the mitochondrial dicarboxylate carrier (DIC; SLC25A10) and the oxoglutarate carrier (OGC; SLC25A11).

Knockdown studies in C2C12 cells demonstrated that DIC mediates succinate transport across the IMM during SDH inhibition and anoxia. This finding prompted a search for a pharmacological inhibitor of DIC. Although butylmalonate was already known to inhibit DIC, its cellular uptake was too slow to effectively prevent succinate transport across the IMM during the critical first minutes of reperfusion. To overcome this limitation, the authors synthesized and tested several butylmalonate derivatives, ultimately identifying diacetoxymethyl butylmalonate (DAB). Incorporation of the acetoxymethyl groups markedly enhanced cellular uptake, after which ubiquitous intracellular esterases rapidly hydrolyzed DAB to release butylmalonate as the active DIC inhibitor. Importantly, a 5-min intravenous administration of DAB in vivo resulted in detectable butylmalonate levels within cardiac mitochondria. Furthermore, intravenous administration of DAB either 5 min before the onset of a 30-min ischaemic period (pre-treatment) or 5 min before the start of reperfusion (post-treatment) reduced infarct size by approximately 50%. These protective effects were associated with either prevention of succinate accumulation during ischaemia (pre-treatment) or reduced utilization of succinate during early reperfusion (post-treatment).^2^

**Figure 1:**
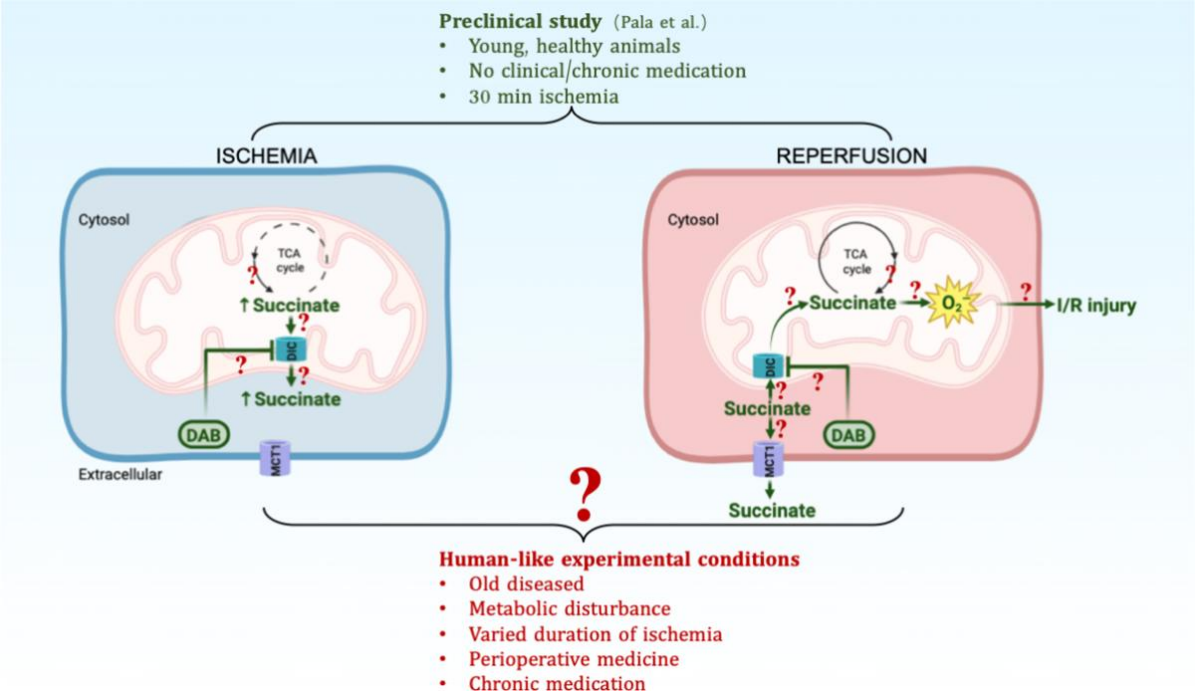
Mitochondrial succinate transport and its modulation by DAB during ischaemia and reperfusion in the healthy heart condition (green symbols), with the unknown effects of human-like conditions (red questions marks) on these mechanisms. Pala *et al*.^2^ shows in young, healthy animals for 30 min ischaemia and without treatments frequently present in clinical conditions of PTCI/CABG, which during ischaemia succinate can accumulate because of its transport from the mitochondrial matrix to the cytosolic compartment through continuous exchange of the mitochondrial exchanger dicarboxylate carrier (DIC). The ischaemic cytosolic succinate accumulation can then drive mitochondrial ROS production during early reperfusion because of its re-entrance through DIC into the mitochondrial matrix. Pala *et al*.^2^ were able to devise a novel compound, DAB) that is quickly taken up by the cardiac cell to inhibit DIC, thereby preventing succinate cytosolic accumulation during ischaemia with pre-ischaemic treatment, or preventing mitochondrial succinate uptake at reperfusion and therefore RET-ROS with pre-reperfusion treatment. Because preclinical discoveries in novel cardioprotective therapies have demonstrated a very poor translatability towards the clinical condition over the last 4 decades of research, the much needed task is now to characterize to what extent this novel protective mechanism remains operative under the most prevalent human-like conditions present during human percutaneous coronary intervention, cardiac surgery or stroke.

The authors are to be congratulated on adding another crucial piece to the succinate–RET–ROS–I/R injury puzzle by (ⅰ) elucidating the transport of succinate between cellular compartments during cardiac I/R and (ⅱ) developing an effective pharmacological intervention targeting this mechanism in vivo (*Figure 1*). The clarity of presentation and the rigour of the biochemical, chemical, and technological approaches employed further strengthen the study, leaving little room for ambiguity or uncertainty.

Nevertheless, the ultimate goal of this body of work is the development of a therapy that can successfully mitigate I/R injury in humans, and this objective should remain central. Numerous cell death pathways contribute to infarct development following an I/R insult, each characterized by distinct temporal activation profiles and varying degrees of dependence on ROS and calcium during reperfusion. Apoptosis, necrosis, and pyroptosis are predominantly active during early reperfusion, whereas ferroptosis and necroptosis mainly contribute to injury during later phases of reperfusion.^4,5^ Succinate-driven RET-mediated ROS production is therefore expected to contribute to only a subset of these pathways, also when one realizes that RET-ROS likely only contributes partly to reperfusion ROS.^6^ Moreover, extensive crosstalk exists among cell death programmes, underscoring the likelihood that targeting a single source of ROS will be insufficient to consistently reduce infarct size or prevent pathological remodelling in the genetically and clinically heterogeneous population of human PCI or stroke patients.

Finally, despite more than four decades of intensive research into cardioprotective mechanisms against I/R injury, translation of these discoveries into clinically effective therapies has been frustratingly disappointing.^7,8^ This failure suggests that a critical element has been missing from our scientific approach to developing protective interventions. One such factor is the pervasive reporting bias in preclinical research, wherein novel drug targets or mechanisms are almost invariably described as successful and protective. Such uniformly positive outcomes are inherently implausible. Under which experimental conditions do these interventions fail? Identifying and reporting these limitations is now essential before advancing yet another cardioprotective strategy into the clinical arena.

A clear example is the prominent report that folic acid administration nearly abolished myocardial infarction in a rat model of cardiac I/R injury.^9^ Subsequent, less prominent but equally important studies demonstrated that protection was completely lost when (ⅰ) ischaemia duration was only slightly extended, (ⅱ) pentobarbital anaesthesia was replaced by more clinically relevant regimens (fentanyl–propofol), or (ⅲ) young animals were replaced by aged or diabetic models.^10^ Anti-oxidant therapy against cardiac I/R is also known to be effective for only short ischaemic periods.^11^ These findings highlight how positive reporting bias remains a major barrier to successful clinical translation.

Accordingly, it is timely to rigorously examine the extent to which the well-established and promising role of succinate-driven RET-ROS pathology in I/R injury remains operative and impactful under clinically relevant conditions, including aging, perioperative medications (heparin, opioids, benzodiazepines, aspirin), varying ischaemic durations, metabolic perturbations (insulin therapy, diabetes, metabolic syndrome), and chronic pharmacological treatments (statins, SGLT2 inhibitors, GLP-1 receptor agonists). These conditions are ubiquitous in the human I/R setting and must be incorporated into future translational studies. It is anticipated that these conditions can affect specific steps in the scheme of mitochondrial succinate transport and RET-ROS-induced I/R injury (*Figure 1*) that reduce the impact of this mechanism on I/R injury. Characterization of these human conditions in experimental settings wherein inhibition of the succinate-driven RET-ROS pathology remains effective should then guide the first clinical trials employing this novel mechanism contributing to I/R pathology. The discovery by Pala *et al*.^2^ of DAB and DIC as crucial components of this novel mechanism can then serve as an important, much needed, stepping stone towards successful clinical translation of I/R injury therapy.
